# Clinical characteristics and outcomes of intracerebral haemorrhage in young vs older adults: insights from the INTERACT3 trial

**DOI:** 10.1093/esj/aakag040

**Published:** 2026-06-30

**Authors:** Maria Khan, Menglu Ouyang, Mohammad Wasay, Lu Ma, Xin Hu, Xiaoying Chen, Laurent Billot, Qiang Li, Paula Muñoz Venturelli, Asita de Silva, Nguyen Huy Thang, Kolawole W Wahab, Jeyaraj D Pandian, Octavio M Pontes-Neto, Carlos Abanto, Antonio Arauz, Chao You, Lili Song, Craig S Anderson, for the INTERACT3 Investigators, Thompson Robinson, J Jaime Miranda, Craig S Anderson, Chao You, Lili Song, Adrian Parry-Jones, Nikola Sprigg, Sophie Durrans, Caroline Harris, Ann Bamford, Olivia Smith

**Affiliations:** Department of Neurology, Rashid Hospital, Dubai, UAE; Department of Neurology, Mohammed bin Rashid University of Medical and Health Sciences, Dubai, UAE; The George Institute for Global Health, Faculty of Medicine, University of New South Wales, Sydney, Australia; Department of Medicine, The Aga Khan University, Karachi, Pakistan; Department of Neurosurgery, West China Hospital, Sichuan University, Chengdu, China; The George Institute for Global Health, Faculty of Medicine, University of New South Wales, Sydney, Australia; Department of Neurosurgery, West China Hospital, Sichuan University, Chengdu, China; The George Institute for Global Health, Faculty of Medicine, University of New South Wales, Sydney, Australia; The George Institute for Global Health, Faculty of Medicine, University of New South Wales, Sydney, Australia; The George Institute for Global Health, Faculty of Medicine, University of New South Wales, Sydney, Australia; The George Institute for Global Health, Faculty of Medicine, University of New South Wales, Sydney, Australia; Clinical Research Center, Faculty of Medicine, Clinica Alemana Universidad del Desarrollo, Santiago, Chile; Clinical Trials Unit, Faculty of Medicine, University of Kelaniya, Colombo, Sri Lanka; Stroke Unit, Gia An 115 Hospital, Ho Chi Minh City, Vietnam; Department of Medicine, University of Ilorin & University of Ilorin Teaching Hospital, Ilorin, Nigeria; Neurology Department, Christian Medical College and Hospital, Ludhiana, India; Department of Neuroscience and Behavioral Sciences, Ribeirão Preto Medical School, University of São Paulo, Ribeirao Preto, Brazil; Cerebrovascular Disease Research Center, National Institute of Neurological Sciences, Lima, Peru; Department of Neurology, Instituto Nacional de Neurologia y Neurocirugia Manuel Velasco Suarez, Mexico City, Mexico; Department of Neurosurgery, West China Hospital, Sichuan University, Chengdu, China; Institute of Science and Technology for Brain-inspired Intelligence, Fudan University, Shanghai, China; Key Laboratory of Computational Neuroscience and Brain-Inspired Intelligence (Fudan University), Ministry of Education, Shanghai, China; The George Institute for Global Health, Faculty of Medicine, University of New South Wales, Sydney, Australia; Institute of Science and Technology for Brain-inspired Intelligence, Fudan University, Shanghai, China; Key Laboratory of Computational Neuroscience and Brain-Inspired Intelligence (Fudan University), Ministry of Education, Shanghai, China; Department of Neurology, Royal Prince Alfred Hospital, Sydney, Australia

**Keywords:** bundled care, ICH, lifestyle risk factors, young stroke

## Abstract

**Introduction:**

ICH is a severe form of stroke with increasing global burden. Although more common in older adults, ICH in younger individuals (≤50 years) is a clinically distinct but understudied subgroup. This secondary analysis of the INTERACT3 trial compared baseline characteristics, management and outcomes between younger and older ICH patients.

**Patients and methods:**

INTERACT3 was a stepped-wedge, cluster-randomised trial conducted in 122 hospitals across 10 countries (2017–2021), evaluating a bundled care intervention for acute ICH. This sub-study analysed 7031 patients and compared demographics, imaging features, in-hospital treatment and 6-month outcomes between age groups. Primary outcomes were functional status (mRS), mortality and quality of life (EQ-5D-3L). Outcomes were analysed using generalised linear-mixed models accounting for clustering by hospital (random effect) and fixed effects for time period and cluster treatment assignment, with additional adjustment for pre-specified patient-level covariates.

**Results:**

Of 7031 patients, 1351 (19.2%) were aged ≤ 50 years. Younger patients were more often male (70.8% vs 62.4%, *P* < .0001), had higher body mass index (BMI) (25.3 vs 23.8 kg/m^2^, *P* < .0001) and were more likely to smoke (36.1% vs 21.4%) and consume alcohol (33.2% vs 13.3%). Despite having slightly larger haematoma volumes (18.0 vs 15.0 mL, *P* < .0001), younger patients had significantly better outcomes, with lower 6-month mortality (9.1% vs 16.6%; adjusted OR 0.42; 95% CI, 0.33–0.54) and reduced rates of death or disability (46.5% vs 57.8%; OR 0.56; 95% CI, 0.48–0.65). A significant age-by-treatment interaction was observed (*P* = .0251).

**Conclusion:**

Younger ICH patients demonstrated a distinct risk profile and better recovery, and benefiting more from bundled care interventions. These findings highlight the importance of early, intensive management and tailored prevention strategies targeting modifiable lifestyle risks in younger populations.

## Graphical Abstract

**Graphical Abstract:**
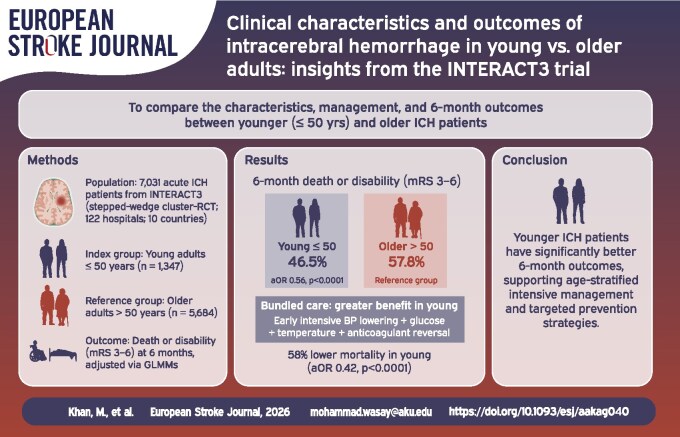

## Introduction

ICH is a life-threatening form of stroke, accounting for 10%–15% of all strokes globally. According to the Global Burden of Disease study, the absolute number of incident cases, deaths and disability-adjusted life years due to ICH has increased over time.^1^ In 2021, ICH caused nearly half of all stroke-related deaths and disability worldwide, highlighting its major contribution to the global stroke burden.^2^ While ICH predominantly affects older adults, it also occurs in individuals under the age of 50—a clinically distinct subgroup that remains understudied despite growing interest in age-related disparities in stroke outcomes. An age threshold of 50 years has been frequently adopted in prior research to distinguish younger-onset ICH, reflecting both epidemiological patterns and differences in underlying risk factor profiles observed across this divide.^3-5^

Emerging evidence suggests that younger ICH patients often present with different aetiologies, risk factor profiles and prognostic trajectories compared to their older counterparts. Even when structural vascular malformations, such as arteriovenous malformations and cavernomas, which are more frequently observed in younger individuals,^3^^,^^6^^,^^7^ are excluded, studies consistently show that younger patients exhibit a distinct risk profile. Commonly implicated risk factors in this age group include hypertension, smoking and alcohol consumption, with hypertension emerging as the predominant modifiable contributor to nontraumatic ICH.^8^

Several studies have also demonstrated that, despite comparable haematoma volumes, younger ICH patients tend to experience more favourable functional outcomes than older adults, likely due to better premorbid health, fewer comorbidities and greater physiological resilience.^9^^,^^10^ However, the clinical profile and management of ICH in younger adults remain poorly characterised, as most large-scale studies have predominantly included older populations. Understanding this subgroup is increasingly important given the growing incidence of hypertension and lifestyle-related risk factors in younger adults. Moreover, ICH in younger patients occurs at the peak of their productive years, carrying substantial long-term socioeconomic and rehabilitation implications. Despite their relatively better outcomes, the burden of disability and the case-fatality rate remains considerable among younger patients, particularly in the presence of coma at admission or elevated ICH scores.^5^^,^^11^

The INTERACT3^12^ study, a large, pragmatic, international, cluster-randomised trial evaluating the effectiveness of a multifaceted care bundle for acute ICH management provides a unique opportunity to investigate age-related differences on a global scale. In this secondary analysis of the INTERACT3 dataset, we aim to compare the clinical characteristics, management practices and outcomes between younger (*<*50 years) and older (>50 years) ICH patients. This age cutoff was selected a priori for this analysis based on both epidemiological and biological rationales. The incidence curve for ICH shifts towards older age groups compared with ischaemic stroke, making younger ICH a clinically and etiologically distinct entity. An age threshold of *<* 50 years enriches for patients with non-degenerative mechanisms and markedly lower prevalence of hypertensive arteriolosclerosis and cerebral amyloid angiopathy, thereby reducing etiological heterogeneity. This threshold is also the most widely used in the existing literature on younger-onset ICH, adopted in the landmark systematic review by Tatlisumak et al.^13^ as well as in multiple registry-based studies and clinical series,^3-5^ enabling meaningful comparison of our findings with prior work. By delineating these differences, we aim to clarify age-dependent presentations of ICH and inform more tailored and effective treatment strategies.

## Patients and methods

### Study design

This is a post-hoc analysis of data from INTERACT3, an international, multicentre, stepped-wedge, cluster-randomised controlled trial conducted between 2017 and 2021 at 122 hospitals across 10 countries (Brazil, Chile, China, India, Mexico, Nigeria, Pakistan, Peru, Sri Lanka and Vietnam). The parent trial evaluated the effectiveness of a goal-directed care bundle for patients with acute spontaneous ICH, focusing on the rapid correction of abnormal physiological variables including blood pressure, glucose, temperature and anticoagulation status.

The hospitals were selected if they did not have any pre-defined protocols for ICH management or if they were using protocols different from those in the study and agreed to switch to the proposed bundle of care. All acute ICH patients presenting at participating sites were registered. Those meeting eligibility criteria were enrolled, while basic information was recorded for those who were excluded. All eligible patients were given information sheets and consent forms. Participants were defined as individuals who provided consent for data collection and 6-month follow-up. The methods have been published earlier^14^ and are briefly described below.

### Patient eligibility criteria

Adults aged 18 years or older were eligible if they presented with an acute stroke syndrome caused by presumed spontaneous ICH, confirmed by clinical history and CT imaging. Patients had to present within 6 h of symptom onset, or within 6 h of the last time they were known to be well if the exact onset was unclear, to be included in the study.

Exclusion criteria included ICH clearly secondary to a structural brain abnormality such as an arteriovenous malformation, aneurysm, tumour, trauma or previous cerebral infarction. Patients were also excluded if the ICH had followed recent thrombolysis or thrombectomy, or if the patients were unlikely to adhere to the study treatment and follow-up.

### Study procedures

For the current sub-study, we compared younger (≤50 years) and older (>50 years) adult patients enrolled in the INTERACT3 trial. Demographic characteristics, imaging findings and hospital management practices were assessed between the 2 age groups.

### Outcomes

Outcomes were assessed at 6 months by blinded central follow-up. For the purpose of this post-hoc analysis, the primary outcome was a composite of death or disability (mRS 3–6). The individual components, death alone and disability alone (mRS 3–5), were also evaluated as additional outcomes of interest. Health-related quality of life was assessed across domains including mobility, self-care, usual activities, pain or discomfort and anxiety or depression, as well as by overall health utility scores.

### Statistical analysis

Descriptive statistics were compared using the Student’s *t*-test for normally distributed continuous variables, Kruskal–Wallis test for skewed continuous variables and chi-squared test for categorical variables. Generalised linear-mixed models were used to assess associations between age group and outcomes. The primary (unadjusted) analysis accounted for clustering by including a random effect for hospital site, a fixed categorical effect for time (4 periods) and a fixed effect for cluster group assignment at each period. In the adjusted model, we additionally included the following pre-specified patient-level covariates: sex, country, pre-stroke mRS, baseline NIHSS, baseline haematoma volume, haematoma location, intraventricular haemorrhage, hypertension, coronary heart disease, prior stroke, diabetes, current smoking status and current alcohol use. The SAS Enterprise Guide (version 8.2) was used for statistical analysis.

### Ethics approval

All procedures followed the ethical standards of the INTERACT3 trial protocol, and informed consent was obtained from all participants or their legal surrogates. The trial was registered on ClinicalTrials.gov (NCT03209258) and the Chinese Clinical Trial Registry (ChiCTR-IOC17011787).

## Results

### Baseline characteristics

A total of 7031 patients were included in this secondary analysis, with 19.2% aged 50 years or younger. Compared to older patients, younger individuals were more often male (70.8% vs 62.4%, *P* < .0001), had higher BMI (25.3 vs 23.8, *P* < .0001) and were more likely to smoke and consume alcohol (both *P* < .0001). They had fewer comorbidities, including hypertension, previous stroke, coronary artery disease, atrial fibrillation and diabetes (all *P* < .01) and demonstrated better pre-stroke functional status (mRS 0 in 84.9% vs 75.4%, *P* < .0001). These younger patients were also less likely to be on antihypertensives, lipid-lowering therapy, antiplatelets or glucose-lowering agents at admission. While systolic BP was comparable, diastolic BP was significantly higher among younger patients (105.7 vs 97.8 mmHg, *P* < .0001). Stroke severity at baseline, assessed by NIHSS and Glasgow Coma Scale (GCS) scores, was similar across age groups. Randomisation between intervention and control groups was balanced (*P* = .4252). These data are shown in Table 1.

**Table 1:** Baseline characteristics of study population.

Baseline characteristics	Overall *n* = 7031	≤50 years *n* = 1347	>50 years *n* = 5684	*P*-value
**Age (years)**				
*n*	7031	1347	5684	<.0001
Mean (SD)	62.0 (12.6)	44.1 (5.8)	66.2 (9.7)	
**Sex**				
Male	4502 (64.0%)	954 (70.8%)	3548 (62.4%)	<.0001
Female	2529 (36.0%)	393 (29.2%)	2136 (37.6%)	
**BMI**				
*n*	5880	1187	4693	<.0001
Mean (SD)	24.1 (3.6)	25.3 (3.9)	23.8 (3.5)	
**Country, *n* (%)**				
China	6353 (90.4%)	1185 (88.0%)	5168 (90.9%)	<.0001
India/Pakistan/Sri Lanka/Vietnam	503 (7.2%)	101 (7.5%)	402 (7.1%)	
Brazil/Peru/Chile/Mexico/Nigeria	175 (2.5%)	61 (4.5%)	114 (2.0%)	
***Past medical history***				
History of hypertension, *n* (%)	4886 (69.5%)	886 (65.8%)	4000 (70.4%)	.0010
Previous stroke, *n* (%)	1075 (15.3%)	116 (8.6%)	959 (16.9%)	<.0001
History of coronary artery disease, *n* (%)	193 (2.7%)	14 (1.0%)	179 (3.1%)	<.0001
History of other heart disease, *n* (%)	254 (3.6%)	26 (1.9%)	228 (4.0%)	.0002
History of atrial fibrillation, *n* (%)	82 (1.2%)	1 (0.1%)	81 (1.4%)	<.0001
History of diabetes mellitus, *n* (%)	726 (10.3%)	87 (6.5%)	639 (11.2%)	<.0001
History of hypercholesterolaemia, *n* (%)	205 (2.9%)	33 (2.5%)	172 (3.0%)	.2597
**Current smoker, *n* (%)**	1359 (19.3%)	338 (25.1%)	1021 (18.0%)	<.0001
**Current alcohol consumption, *n* (%)**	1394 (19.8%)	344 (25.5%)	1050 (18.5%)	<.0001
**mRS score of 0 before onset, *n* (%)**	5380 (77.2%)	1134 (84.9%)	4246 (75.4%)	<.0001
***Medication at admission***				
Antihypertensive medication, *n* (%)	3070 (43.7%)	469 (34.8%)	2601 (45.8%)	<.0001
Blood glucose lowering agents, *n* (%)	510 (7.3%)	60 (4.5%)	450 (7.9%)	<.0001
Statin or another lipid lowering agent, *n* (%)	221 (3.1%)	19 (1.4%)	202 (3.6%)	<.0001
Aspirin or another antiplatelet agent, *n* (%)	377 (5.4%)	31 (2.3%)	346 (6.1%)	<.0001
Anticoagulation agent, *n* (%)	65 (0.9%)	6 (0.4%)	59 (1.0%)	.0410
**Systolic blood pressure (mmHg)**				<.0001
*n*	7029	1346	5683	
Mean (SD)	174.5 (28.3)	176.0 (30.9)	174.2 (27.6)	
**Diastolic blood pressure (mmHg)**				<.0001
*n*	7029	1346	5683	
Mean (SD)	99.3 (17.8)	105.7 (20.3)	97.8 (16.9)	
**NIHSS at admission**				.4220
*n*	6839	1309	5530	
Median (Q1, Q3)	13.0 (7.0, 22.0)	13.0 (7.0, 23.0)	13.0 (7.0, 22.0)	
**GCS score**				.7779
*n*	7024	1346	5678	
Median (Q1, Q3)	12.0 (9.0, 14.0)	12.0 (9.0, 14.0)	12.0 (9.0, 14.0)	
**Randomized group, *n* (%)**				
Intervention	3220 (45.8%)	630 (46.8%)	2590 (45.6%)	.4252
Control	3811 (54.2%)	717 (53.2%)	3094 (54.4%)	

Data are *n* (%), mean (SD) or median (IQR).

Descriptive statistics were with the *t*-test for normally distributed data, Kruskal–Wallis test for skewed continuous variables and chi-squared test for categorical variables.

Abbreviations: BMI = body mass index; GCS = Glasgow Coma Scale.

**Figure 1:**
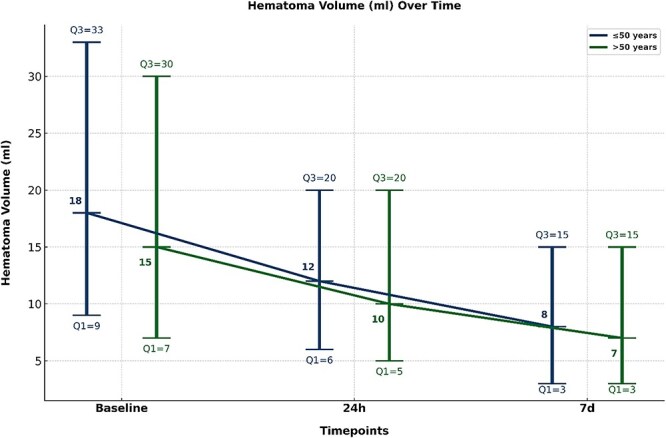
Median haematoma volumes in millilitre with interquartile range at baseline, 24 h and day 7 for each age group.

At baseline, younger patients had larger median haematoma volumes than older patients (18.0 vs 15.0 mL, *P* < .0001). Haematomas were more often midline (7.0% vs 5.4%) and deep (84.7% vs 81.7%) in younger patients, while cerebellar (2.3% vs 6.1%) and brainstem involvement (6.7% vs 4.7%) were less frequent. Intraventricular extension was also less common in the young (25.8% vs 30.7%). No significant differences were seen for side or cortical location. At 24 h, median volume remained slightly higher in younger patients (12.0 vs 10.0 mL), converging by day 7 (*P* = .97) (Figure 1).

### Hospital management

Table 2 relates to management aspects during the hospital stay. At admission, most patients were admitted under neurosurgery (73.4%), with younger patients slightly less likely to be admitted to neurosurgery compared to older patients (70.2% vs 74.1%) and intensive care admissions were marginally higher in younger patients (13.4% vs 11.2%; *P* = .028).

**Table 2:** Comparison of hospital management between younger (≤50 years) and older patients (>50 years).

Baseline characteristics	Overall *n* = 7031	≤50 years *n* = 1347	>50 years *n* = 5684	*P*-value
**Admission department to hospital, *n* (%)**				.0283
Neurosurgery	5157 (73.4%)	946 (70.2%)	4211 (74.1%)	
Neurology	515 (7.3%)	101 (7.5%)	414 (7.3%)	
Intensive care	814 (11.6%)	180 (13.4%)	634 (11.2%)	
Emergency department	310 (4.4%)	73 (5.4%)	237 (4.2%)	
Others	234 (3.3%)	47 (3.5%)	187 (3.3%)	
***Management in the first 24 h***				
BP lowering treatment in the first 24 h, *n* (%)	5468 (77.8%)	1089 (80.8%)	4379 (77.1%)	.0029
Intensive treatment for glucose control in the first 24 h, *n* (%)	510 (7.3%)	60 (4.5%)	450 (7.9%)	<.0001
Oral agents for glycaemic control in the first 24 h, *n* (%)	413 (81.0%)	47 (78.3%)	366 (81.3%)	.5781
Insulin treatment for glycaemic control in the first 24 h, *n* (%)	128 (25.1%)	15 (25.0%)	113 (25.1%)	.9851
Pyrexia treatment for glycaemic control in the first 24 h, *n* (%)	594 (8.5%)	157 (11.7%)	437 (7.7%)	<.0001
***Management for 2–7 days***				
Intravenous BP lowering, *n* (%)	4674 (67.2%)	969 (72.6%)	3705 (65.9%)	<.0001
Oral BP lowering, *n* (%)	4812 (69.2%)	921 (69.0%)	3891 (69.2%)	.8817
Insulin, *n* (%)	938 (13.5%)	166 (12.4%)	772 (13.7%)	.2130
Hypothermia, *n* (%)	1420 (20.4%)	290 (21.7%)	1130 (20.1%)	.1849
PCC administered, *n* (%)	621 (8.9%)	130 (9.7%)	491 (8.7%)	.2465
Fresh frozen plasma, *n* (%)	165 (2.4%)	41 (3.1%)	124 (2.2%)	.0616
Vitamin K administrated, *n* (%)	336 (4.8%)	92 (6.9%)	244 (4.3%)	<.0001
Decompressive surgery	1860 (26.5%)	442 (33.1%)	1418 (25.2%)	<.0001
Craniotomy	1035 (55.6%)	267 (60.4%)	768 (54.2%)	
Craniectomy	46 (2.5%)	8 (1.8%)	38 (2.7%)	
Endoscopy	68 (3.7%)	18 (4.1%)	50 (3.5%)	
Aspiration/Intraventricular drainage/Catheterization or other	711 (38.2%)	129 (29.2%)	562 (39.6%)	
Mechanical ventilation, *n* (%)	1451 (20.8%)	333 (24.9%)	1118 (19.9%)	<.0001
Intensive care admission, *n* (%)	2538 (36.4%)	590 (44.2%)	1948 (34.6%)	<.0001
Assisted feeding, *n* (%)	3666 (52.6%)	726 (54.3%)	2940 (52.2%)	.1627
Decision to withdraw active care, *n* (%)	47 (0.7%)	6 (0.4%)	41 (0.7%)	.2625

Data are *n* (%), mean (SD) or median (IQR).

Descriptive statistics were with the *t*-test for normally distributed data, Kruskal–Wallis test for skewed continuous variables and chi-squared test for categorical variables.

Abbreviations: BP = blood pressure; PCC = prothrombin complex concentrate.

Blood pressure-lowering treatment was started within the first 24 h for 77.8% of patients overall, and was more frequently used in younger patients (80.8% vs 77.1%; *P* = .0029). While the use of oral agents and insulin for management of hyperglycaemia did not differ between younger and older age groups, intensive glucose control was less often initiated in younger individuals (4.5% vs 7.9%; *P* < .0001) in the first 24 h. However, treatment for pyrexia was more common among younger patients during this initial period (11.7% vs 7.7%; *P* < .0001).

Between days 2 and 7, younger patients were more likely to receive intravenous blood pressure management (72.6% vs 65.9%, *P* < .001) and vitamin K administration (6.9% vs 4.3%, *P* < .001). Other aspects of medical management, including oral antihypertensives, insulin use, hypothermia therapy, prothrombin complex concentrate and fresh frozen plasma, were largely similar between groups.

Decompressive surgery was performed more frequently in younger patients (33.1% vs 25.2%; *P* < .0001), with craniotomy being the predominant surgical approach in both groups. Rates of craniectomy, endoscopic procedures and catheter-based drainage were broadly comparable, although younger patients were less likely to undergo aspiration or intraventricular drainage (29.2% vs 39.6%).

Mechanical ventilation (24.9% vs 19.9%; *P* < .0001) and intensive care admission (44.2% vs 34.6%; *P* < .0001) were significantly more common in younger patients. Decisions to withdraw active care were rare overall, with no significant difference by age (0.4% vs 0.7%; *P* = .2625).

### Outcomes

Younger patients had better functional outcomes at 6 months than older patients. They were more likely to be symptom-free (mRS = 0: 14.0% vs 8.3%; adjusted OR 0.51; 95% CI, 0.45–0.58; *P* < .0001) and had lower rates of death or disability (mRS 3–6: 46.5% vs 57.8%; adjusted OR 0.56; 95% CI, 0.48–0.65; *P* < .0001). Mortality at 6 months was substantially lower among younger patients (9.1% vs 16.6%; adjusted OR 0.42; 95% CI, 0.33–0.54; *P* < .0001), and disability alone, defined as mRs 3–5, was also less frequent (41.1% vs 49.0%; adjusted OR 0.64; 95% CI, 0.55–0.74; *P* < .0001).

Importantly, a statistically significant interaction between age group and treatment effect was observed for the primary outcome of death or disability (mRS 3–6) (*P* for interaction = .0251) and for disability alone (mRS 3–5) (*P* for interaction = .0085), indicating that the magnitude of treatment effect differed across age strata. Stratified analyses demonstrated that the intervention effect was more pronounced in patients aged *<* 50 years, whereas the corresponding effect in the older age group was attenuated. These findings were consistent across adjusted models. No significant age-by-treatment interactions were observed for mortality alone, recurrent events or other secondary outcomes.

Across quality-of-life domains, younger patients reported fewer difficulties with usual activities (48.2% vs 38.9%; adjusted OR 0.59; 95% CI, 0.51–0.68; *P* < .0001) and mobility (73.4% vs 62.8%; adjusted OR 0.53; 95% CI, 0.45–0.62; *P* < .0001). Self-care was also better among younger patients (61.4% vs 54.1% reporting no problems; adjusted OR 0.61; 95% CI, 0.52–0.70; *P* < .0001). No significant differences were observed between age groups for pain or discomfort (*P* = .82) or anxiety and depression (*P* = .56). Mean overall health utility scores were higher in younger patients (0.7 vs 0.6; adjusted mean difference 0.11; 95% CI, 0.09–0.14; *P* < .0001; Table 3).

**Table 3:** Comparison of 6-month outcomes between younger (≤50 years) and older patients (>50 years).

Outcomes	Overall	≤50 years	>50 years	Unadjusted ^a^				Adjusted ^b^			
mRS at 6 months, *n* (%)				*n*	Odds ratio	*P*-value	ICC	*n*	Adjusted odds ratio	*P*-value	ICC
No symptoms	589 (9.4%)	167 (14.0%)	422 (8.3%)	6250	0.54 (0.48, 0.61)	<.0001	0.055	5907	0.51 (0.45, 0.58)	<.0001	0.027
No significant disability	1801 (28.8%)	397 (33.2%)	1404 (27.8%)								
Slight disability	383 (6.1%)	75 (6.3%)	308 (6.1%)								
Moderate disability	1118 (17.9%)	257 (21.5%)	861 (17.0%)								
Moderate severe disability	784 (12.5%)	116 (9.7%)	668 (13.2%)								
Severe disability	598 (9.6%)	73 (6.1%)	525 (10.4%)								
Death	977 (15.6%)	110 (9.2%)	867 (17.2%)								
**Death or disability (mRS 3–6) at month 6**	3477 (55.6%)	556 (46.5%)	2921 (57.8%)	6250	0.6 (0.53, 0.69)	<.0001	0.039	5907	0.56 (0.48, 0.65)	<.0001	0.024
**Death at month 6, *n* (%)**	977 (15.6%)	110 (9.2%)	867 (17.2%)	6435	0.47 (0.37, 0.58)	<.0001	0.076	6080	0.42 (0.33, 0.54)	<.0001	0.039
**Disability (mRS 3–5) at month**	2500 (47.4%)	446 (41.1%)	2054 (49.0%)	5273	0.69 (0.60, 0.80)	<.0001	0.027	4993	0.64 (0.55, 0.74)	<.0001	0.023
***Health-related quality of life of the EQ-5D-3L*** **Usual activities**											
No problem	2152 (40.8%)	523 (48.2%)	1629 (38.9%)	5273	0.64 (0.57, 0.73)	<.0001	0.026	4993	0.59 (0.51, 0.68)	<.0001	0.031
Some problems	1594 (30.2%)	332 (30.6%)	1262 (30.1%)								
Extreme problem	1527 (29.0%)	230 (21.2%)	1297 (31.0%)								
**Mobility**				5273	0.57 (0.49, 0.66)	<.0001	0.021	4993	0.53 (0.45, 0.62)	<.0001	0.014
No problem	3427 (65.0%)	796 (73.4%)	2631 (62.8%)								
Some problems	1100 (20.9%)	201 (18.5%)	899 (21.5%)								
Extreme problem	746 (14.1%)	88 (8.1%)	658 (15.7%)								
**Pain or discomfort**				5273	1.03 (0.88, 1.19)	.7356	0.024	4993	0.98 (0.84, 1.15)	.8215	0.010
No problem	3681 (69.8%)	757 (69.8%)	2924 (69.8%)								
Some problems	1546 (29.3%)	319 (29.4%)	1227 (29.3%)								
Extreme problem	46 (0.9%)	9 (0.8%)	37 (0.9%)								
**Anxiety or depression**				5273	1.00 (0.85, 1.18)	.9801	0.042	4993	0.95 (0.80, 1.13)	.5649	0.026
No problem	4090 (77.6%)	844 (77.8%)	3246 (77.5%)								
Some problems	1107 (21.0%)	226 (20.8%)	881 (21.0%)								
Extreme problem	76 (1.4%)	15 (1.4%)	61 (1.5%)								
**Self-care**				5273	0.67 (0.59, 0.77)	<.0001	0.022	4993	0.61 (0.52, 0.70)	<.0001	0.016
No problem	2931 (55.6%)	666 (61.4%)	2265 (54.1%)								
Some problems	1164 (22.1%)	254 (23.4%)	910 (21.7%)								
Extreme problem	1178 (22.3%)	165 (15.2%)	1013 (24.2%)								
**Mean overall health utility score**				6250	0.12 (0.09, 0.14)	<.0001	0.070	5907	0.11 (0.09, 0.14)	<.0001	0.035
*n*	6250	1195	5055								
Mean (SD)	0.6 (0.4)	0.7 (0.3)	0.6 (0.4)								

^a^Un-adjusted analysis: logistic regression for death/disability with a random effect for cluster (hospital site), a fixed effect indicating the group assignment of each cluster at each step and a fixed categorical effect of 6-month interval.

^b^Model-adjusted baseline characteristics including sex, baseline NIHSS and country, mRS before stroke, sex, baseline haematoma volume, location of haematoma, intraventricular haemorrhage, hypertension, coronary heart disease, previous history of stroke, diabetes, current smoker and current alcohol user.

Abbreviations: aOR = adjusted odds ratio; ICC = intraclass correlation coefficient; OR = odds ratio.

## Discussion

In this secondary analysis of the INTERACT3 trial, patients 50 years of age or younger with ICH had significantly better functional outcomes and lower mortality at 6 months compared to older patients, despite having larger baseline haematoma volumes. They also derived greater benefit from the early, intensive care bundle (focused on blood pressure, glucose, temperature and anticoagulation reversal), particularly in reducing death or disability, and showed a distinct risk profile marked by more lifestyle-related factors, while older patients had a higher burden of vascular comorbidities.

This age-related contrast in risk profiles, where younger patients exhibited a higher prevalence of smoking, alcohol consumption and a higher BMI, while older patients had a greater prevalence of hypertension, diabetes and coronary artery disease, is supported by other studies. A study from China^9^ showed similar risk differences among younger vs older patients, with fewer younger patients having hypertension, diabetes and prior stroke but more having higher BMI. Another study also found lifestyle factors like smoking and obesity to be associated with non-lesional spontaneous ICH in young patients.^15^ Excessive alcohol consumption and high BMI were risk factors identified in another study by Yang et al.^10^ Consistent with these findings, the INTERSTROKE study identified binge alcohol drinking as a significant risk factor for ICH in individuals younger than 45 years of age (OR 4.06; 95% CI [1.27–13.0]).^16^ This study also found hypertension as a significant risk factor in the young.

These findings suggest that the underlying pathophysiology of ICH may differ by age, with modifiable lifestyle factors playing a greater role in younger patients and cumulative vascular injury predominating in older individuals. From a prevention standpoint, these observations emphasise the need for targeted strategies: promoting lifestyle modifications among younger adults and aggressive vascular risk factor control in older populations.

Our findings also suggest a more aggressive and intervention-focused management approach in younger ICH patients. Despite being slightly less likely to be admitted under neurosurgery, younger patients had higher rates of early blood pressure lowering, intensive care admission and decompressive surgery. Younger age was a predictor for ICU admission in the INTERACT2 trial as well.^17^ All these measures along with higher use of pyrexia treatment and vitamin K administration, along with more frequent mechanical ventilation, indicating greater therapeutic optimism in this group. Older patients, while more likely to receive aspiration precautions or intraventricular drainage, were managed more conservatively overall. These differences likely reflect both clinical judgement regarding prognosis and a higher threshold for withdrawal of care in younger individuals. Younger patients also face less of this therapeutic nihilism as suggested by previous studies.^18^

In our study, younger patients (≤50 years) consistently demonstrated better outcomes following ICH, with lower 6-month mortality (9.2% vs 17.2%), lower rates of disability and higher likelihood of achieving no symptoms or no significant disability (33.2% vs 27.8%) compared to older patients. These findings align closely with prior literature, where short-term mortality among young ICH patients ranged from 8% to 20% and favourable functional outcomes (mRS 0–2) were reported in approximately 48%–60% of cases.^3^^,^^5^^,^^6^^,^^10^

Younger patients also demonstrated better health-related quality of life. These findings align with previous studies demonstrating that younger individuals often recover more favourably after stroke, likely due to factors such as fewer comorbidities, greater vascular integrity and enhanced neuroplasticity.^19^^,^^20^ Interestingly, despite presenting with slightly larger haematoma volumes at baseline, younger patients had superior recovery, suggesting that haematoma size is not the sole determinant of prognosis. Instead, intrinsic physiological resilience and neurological reserve appear to play a critical role in mediating recovery potential. Supporting this, in a study by Koivunen et al.,^4^ elderly patients had haematoma volume that predicted 3-month mortality, a relationship not observed in younger individuals, further underscoring the age-dependent influence of systemic and neurological factors on outcomes.

Beyond baseline differences, management strategies during hospitalisation may have also influenced outcomes. Younger patients were more likely to receive early blood pressure lowering treatment within the first 24 h of admission. This more intensive approach could reflect a clinical tendency to intervene more aggressively in younger patients, given their greater potential for long-term recovery. It is also plausible that younger individuals tolerated intensive haemodynamic management better, minimising the risk of secondary injury. Early control of blood pressure may thus have played a contributory role in improving outcomes in this group. Our findings also align with evidence from the INTERACT2 trial, which emphasised the benefit of early blood pressure control in improving outcomes, particularly when implemented promptly.^21^

Importantly, a significant interaction between age group and treatment effect was observed for both death or disability (mRS 3–6) and disability alone (mRS 3–5), indicating that the effectiveness of the intervention differed by age. No evidence of interaction was found for other clinical outcomes or health-related quality-of-life domains, suggesting that while functional recovery varied according to age, subjective experiences such as pain, anxiety and depression were influenced by broader psychosocial or environmental factors rather than the intervention itself.

### Public health implications

These findings highlight the importance of population-level interventions to reduce the burden of ICH. Multifaceted strategies are needed: aggressive detection and control of hypertension, through community-based screening, which should start at younger ages, affordable antihypertensive therapy and strengthened primary care services; taxation and public education campaigns to curb excessive alcohol consumption and smoking and initiatives promoting healthy weight maintenance and physical activity. Implementing these measures in both high- and low-resource settings can help mitigate the rising global burden of ICH, particularly as younger adults present with lifestyle-related risk factors while older adults accumulate vascular comorbidities.

### Limitations

Several limitations should be acknowledged. Despite adjustment for multiple confounders, residual confounding related to unmeasured factors such as socioeconomic status, health-seeking behaviours and genetic differences cannot be ruled out. The definition of “younger” as ≤ 50 years, although pragmatic, may not fully capture the biological heterogeneity within this group. Additionally, management and rehabilitation practices may have varied across participating centres, and this could have contributed to outcome variability.

Generalisability is also a consideration: although the INTERACT3 trial^12^ enrolled patients across 10 countries, 90.4% of the cohort was recruited from China. South and Southeast Asian countries (India, Pakistan, Sri Lanka and Vietnam) contributed 7.2%, while Latin American and African sites (Brazil, Peru, Chile, Mexico and Nigeria) contributed only 2.5%. There were meaningful regional differences in baseline risk factor profiles with notable differences in the prevalence of BMI, hypertension, diabetes and smoking across the 3 regions. Additionally, the subgroup analysis of the primary outcome by region showed a significant treatment interaction across regions, with the intervention effect markedly stronger in the Latin American and African group than in China. These regional differences in patient characteristics and treatment response should be considered when interpreting the findings of this sub-study, which predominantly reflects the Chinese ICH population. The generalisability of these age-related observations to other ethnic groups, healthcare settings and regions with differing risk factor burdens warrants caution. Lastly, subgroup analyses inherently carry the risk of overinterpretation, and the findings should therefore be considered hypothesis-generating.

## Conclusion

Younger patients with ICH often present with a distinct risk profile, characterised by modifiable lifestyle-related factors such as higher rates of alcohol consumption and elevated BMI, which may contribute to disease onset but also offer opportunities for targeted prevention. Younger ICH patients also experienced significantly better outcomes at 6 months despite having higher haematoma volumes. This may suggest that early intensive management, particularly blood pressure lowering, is especially effective in improving outcomes in younger individuals. Future research should aim to validate these findings in larger cohorts and explore targeted strategies that address the specific needs and risk profiles of younger patients with ICH.

## Supplementary Material

Supplemental_Materials_INTERACT3_investigators_aakag040

## Data Availability

Individual, de-identified participant data used in these analyses can be shared on request from any qualified investigator after the approval of a protocol and signed data access agreement via both the trial steering committee and the research office of The George Institute for Global Health, Sydney, Australia.
