# Supplementary material for: Clinical characteristics and outcomes of intracerebral haemorrhage in young vs older adults: insights from the INTERACT3 trial
Source: Eur Stroke J. 2026 Jun 30;11(6):aakag040. doi: 10.1093/esj/aakag040 (PMC13317946; doi:10.1093/esj/aakag040)
Supplement: Supplemental_Materials_INTERACT3_investigators_aakag040 [file supplemental_materials_interact3_investigators_aakag040.docx]

**Supplemental Materials**

**Predictive accuracy of physician estimates of death and recovery after acute intracerebral hemorrhage: pre-specified analysis in INTERACT3 study**

Menglu Ouyang, et al.

**1. List of INTERACT3 study group and trial investigators**

***Trial Steering Committee***

Professor Thompson Robinson (Chair, independent), University of Leicester, Leicester, UK

Professor J. Jaime Miranda (Deputy Chair, independent), Universidad Peruana Cayetano

Heredia, Lima, Peru

Professor Craig S. Anderson (Co-Principal Investigator), The George Institute for Global

Health Australia and China

Professor Chao You (Co-Principal Investigator), West China Hospital, Chengdu, China

Dr Lili Song (Associate Investigator, Global Project Lead), The George Institute for Global

Health China

Professor Adrian Parry-Jones (member, independent), University of Manchester, Manchester,

UK

Professor Nikola Sprigg (member, independent), University of Nottingham, Nottingham, UK

Ms Sophie Durrans (observer, 2019-2021), Department of Health and Social Care, London, UK

Ms Caroline Harris (observer, 2021-2023), Department of Health and Social Care, London, UK

Ms Ann Bamford (patient representative), UK

Ms Olivia Smith (patient representative), UK

***Data Safety Monitoring Board***

Professor Robert Herbert (Chair), University of New South Wales, Sydney, Australia

Professor Christopher Chen, National University Hospital, Singapore

Professor William Whiteley, University of Edinburgh, Edinburgh, UK

Professor Rong Hu, Southwest Hospital, Chongqing, China

***Statisticians***

Professor Laurent Billot, The George Institute for Global Health, Australia

Mr Qiang Li, The George Institute for Global Health, Australia

Ms Jayanthi Mysore, The George Institute for Global Health, Australia

***Medical Review Committee***

Associate Professor Xin Hu, West China Hospital, Chengdu, China

Dr Yao Zhang, Shenyang First People’s Hospital, Shenyang Brain Institute, Shenyang, China

Dr Feifeng Liu, Shanghai East Hospital, Tongji University, Shanghai, China

***Imaging Adjudication Committee***

Dr Yuki Sakamoto, Graduate School of Medicine, Nippon Medical School, Tokyo, Japan

Dr Shoujiang You, The Second Affiliated Hospital of SooChow University, Jiangsu, China

Dr Qiao Han, Suzhou Hospital of Traditional Chinese Medicine, Suzhou, China

Dr Bernard Crutzen, Cliniques Universitaires Saint-Luc, Brussels, Belgium

Dr Yunke Li, The George Institute for Global Health China, Beijing, China

Dr Emily Cheung, Neurology Department, Royal Prince Alfred Hospital, Sydney, Australia

***Process Evaluation & Economic Evaluation Committee***

Professor Stephen Jan, The George Institute for Global Health, Australia

Dr Hueiming Liu, The George Institute for Global Health, Australia

Dr Menglu Ouyang, The George Institute for Global Health, Australia

Dr Lingli Sun, The George Institute for Global Health China, Beijing, China

Dr Honglin Chu, Research Center of Clinical Epidemiology, Peking University Third Hospital, Beijing, China

Anila Anjum, Clinical Trials Unit, The Aga Khan University, Karachi, Pakistan

Francisca Gonzalez Mc Cawley, Centro de estudios Clínicos, Instituto de Ciencias e Innovación en Medicina. Facultad de Medicina, Universidad del Desarrollo, Chile

***Follow-up Centre***

*China:* Shanghai – Yan Wu (Coordinator), Lingling Feng, Jingjing Ni, Caixiu Du, Weiwei Fu

*Chile:* Centro de Estudios Clínicos, Instituto en Ciencias e Innovación en Medicina, ICIM,

Facultad de Medicina- Clínica Alemana, Universidad del Desarrollo - Alejandra del Rio

*Brazil:* Hospital das Clinicas da Facultade de Medicina de Riberao Pretto – Bruna Rimoli,

Rodrigo Cerantola

*Sri Lanka:* RemediumOne Ptd Ltd *-* Thanushanthan Jeevarajah, Madhushani Kannangara,

Andrene Joseph, Chamath Nanayakkara

***International Coordinating Centre (ICC) - The George Institute for Global Health***

*Project Management* – Xiaoying Chen, Alejandra Malavera, Chunmiao Zhang, Zhao Yang,

Brook Li, Zhuo Meng, Menglu Ouyang; *Data Management and Programming* – Leibo Liu, Yi Ning, Le Dong, Manuela Armenis; *Quality Assurance* - Joyce Lim, Helen Monaghan.

***Regional Coordinating Centres (RCCs)***

*West China Hospital and The George Institute for Global Health (China):* Lu Ma, Xin Hu, Xi Li, Rui Luo, Guojuan Cheng, Yilin Dong, Ziqin Liu, Shuihong Wang, Ying Zhang, Jipeng

Cheng, Hui Shi, Wenjing Li, Langming Mou, Ping Yi, Chen Chen, Xue Chen

*RemediumOne Pvt Ltd (Sri Lanka):* ShalomiWeerawardena (Clinical Research Associate),

Poornima Ellawala (Clinical Research Associate), Enalee Ranasinghe (Clinical Research

Associate), Chrishmi Rodrigo (Clinical Research Associate).

*Horus Co, LTd (Vietnam):* Uyen Hong Ha (Project Manager), Linh Le Thi My (Project

Manager), Yen Mai Bao (Clinical Research Associate), Duong Hoang Thi Thuy (Clinical

Research Associate), Thu Nguyen Hoang (Study Coordinator), Hai Ngo Thanh (Clinical

Research Associate).

*University of Ilorin Teaching Hospital (Nigeria):* KolawalaWahab (National Leader), Sunday

Adeniyi (Project Manager).

*Christian Medical College and Hospital, Ludhiana (India):* Jeyaraj Pandian (National Leader), Megha Khanna (Project Manager).

*Centro de Estudios Clínicos, Instituto en Ciencias e Innovación en Medicina, ICIM, Facultad*

*de Medicina- Clínica Alemana, Universidad del Desarrollo (Chile):* Paula Muñoz Venturelli

(National Leader), Francisca González (Project Manager), Francisca Urrutia Goldsack (Clinical Research Associate), Alejandra Del Río (Clinical Research Associate).

*The Aga Khan University (Pakistan):* Mohammad Wasay (National Leader), Dilshad Begum

(Project Manager), Anila Anjum (Study Coordinator).

***INTERACT3 Principal Investigators and Coordinators (centre, with numbers of patients in***

***parentheses)***

***Brazil***

*Hospital das Clinicas da Facultade de Medicina de Riberao Pretto (23):* Octavio Pontes-Neto, Millene Camilo, Francisco Dias, Octavio Vincenzi, Rodrigo Cerantola; *Neurológica –*

*Neurologista e Neurocirurgião em Joinville SC (6):* Carla Moro, Renata Santos, Nara Texeira, Alexandre Longo, Rafaela Liberato; *Hospital de Clínicas de Porto Alegre (5):* Sheila Martins, Arthur Pille, Bruna Chwal, Isabel Silva, Natacha Titton; *Moinhos de Vento (1)*: GustavoWeiss, Daissy Mora, Magda Martins, Leonardo Carbonera; *Faculty of Medicine of Botucatu, UNESP (1):* Rodrigo Bazan, Gabriel Modolo, Fernanda Winckler, Luana Miranda, Juli Souza.

***Chile***

*Hospital Clinico Herminda Martin (Hospital de Chillan) (15):* Alexis Rojo, Wilhelm Uslar,

Lorena Medel; *Clínica Alemana de Santiago (8):* Paula Munoz Venturelli, Javiera Lopez, Diego Herrero, Pablo Lavados, Barbara Vargas Latorre; *Hospital Base San José de Osorno (7):*

Nathalie Conejan, Tomas Esparza, Patricio Sotomayor; *Hospital Carlos Van Buren (6):*

Denisse Wenger, Juan Pablo Gigoux, Aldo Letelier, Lilian Acevedo, Vivianne Moya; *Complejo Asistencial Dr. Victor Rios Ruiz (Hospital de Los Angeles) (4):* Cristian Figueroa, Nicol. Vallejos; *Hospital Santiago Oriente Dr. Luis Tisne (4):* Rodrigo Guerrero, Mauricio

Velasquez; *Hospital Metropolitano La Florida (4):* Jose Vallejos, Kimerly Pallauta, Tamara

Santibanez, Angelo Queirolo, Andrea Lobos.

***China***

*Suining Central Hospital (224):* Yongming Jiang, Weimin Li, Wei Huang, Ke Luo, Gangying

Liu; *Liaoning Thrombus Disease Treatment Centre Combing TCM and Western Medicine (205):* Guanghai Tang, Guang Yang, Hongtao Jiang, Xu Zhang, Hongyan Jing; *Dazhu County People’s Hospital (200):* Sheng Zhu, Bo Pu, Dong Lv, Hui Kang, Qiuping Hu; *Jiangsu Rudong County People's Hospital (200):* Xiaochun She, Xiaoming Jiang, Yanli Chen, Shenghua Yang, Jianjun He; *Mianyang Central Hospital (165):* Zongping Li, Gang Cheng, Hailin Huang, Xiaoyi Wang, Jianqiong Lin; *Zigong Fourth People’s Hospital (162):* Minhui Chen, Chenghao Yang, Hao Ding, Yunliang Deng, Fei Luo; *No.987 Hospital of Joint Logistic Support Force (162):* Rongjun Zhang, Xiaofeng Wang, Hongbing Zhang, Xiaoliang Yang, Yang Zhang; *Dayi County People’s Hospital (160):* Chengyi Yang, Yu He, Feng Liu, Rongjie Wang, Yuhui Zhang; *The First People’s Hospital of Yuanping (160): Xiaodong Xin, Bin Feng, Wanru Hao, Chang Song, Yun Guo; Xuzhou Central Hospital (160):* Dehua Jiang, Jie Chen, Changtong Tang, Hongliang Zhu; *The First People’s Hospital of Shenyang (159*)*:* Xin Li, Jin Cui, Haidong Xu, Boyang Li, Fusheng Tang; *Chongzhou People’s Hospital (153):* Yuanbin Li, Min Gao, Bo Yang; *Chengdu Second People’s Hospital (146):* Xuejun Xu, Bing Deng, Yi Zheng, Yuanhong Ge, Keyu Chen; *The Third Hospital of Mianyang (142):* Yang Liu, Xinshen Li, Tingting Zhong, Jianfeng Xu, Hai Zhang; *Liaocheng People's Hospital (142):* Jiyue Wang, Jianxin Zhu, Hanyu Sun, Fuhua Yu, Xueguang Zhang; *West China Hospital Sichuan University (126):* Chao You, Lu Ma, Xin Hu, Jianguo Xu, Xi Li; *The Second People’s Hospital of Neijiang (121):* Mingsen Zhang, Bin Wang, Yiming Ma, Donglin Jiang, Jun Zhou; *The First People’s Hospital of Neijiang (120):* Cong Liu, Wenhong Nie, Mingguo Li, Tao Tian, Yong Li; *Guangyuan Central Hospital (120):* Mingfang He, Xiaolong Tu, Zhengjun Wu, Hong Liu, Dongsheng Zhong; *Tianjin Medical University Central General Hospital (120):* Rongcai Jiang, Jian Sun, Ye Tian, Yingsheng Wei, Shuo An; *Mianzhu People’s Hospital (118):* Pingbo Wei, Le Luo, Bin Lin, Gang Liu, Yan Wen; *Renmin Hospital of Wuhan University (116):* Qiang Cai, Qianxue Chen, Pan Lei, Zhiyang Li, Meifang Zhang; *Nanchong Central Hospital (113):* Jiaquan He, Yan Chen; *The First Hospital of Kunming (112):* Jun Liu, Xinghai Liu, Junyan Li, Min Chen, Jing Wang;

*Xinhua Hospital of Zhejiang Province (112):* Bingzhi Zhou, Baichun Ye, Jiancheng Zhang,

Manyuan Zhang, Xuming Pan; *Wuhan Third Hospital-Tongren Hospital of Wuhan University*

*(109):* Xiaoxiang Yu, Jian Xu, Qingbao Xiao, Yuefei Wang, Liang Tao; *Dezhou People's*

*Hospital (93):* Lin Shi; *The People's Hospital of Leshan (91):* Niandong Zheng, Guoliang You, Bo Lei, Shu Chen, Honggang Wu; *Huashan Hospital of Fudan University Neurosurgery Department (88):* Jin Hu, Jianlan Zhao, Jian Yu, Qiang Yuan, Zhuoying Du; *Santai County People’s Hospital (84):* Xielin Tang, Qianke Li, Shenghua Liu, Feilong Yang, Kui Xiao; *Chongqing Traditional Chinese Medicine Hospital (84):* Chao Luo, Guang Wang, Xudong Che, Zhipeng Teng, Wenwu Wan; *The Central Hospital of Wuhan (84):* Jun Li, Yu Liu, Mingbo Fan, Tao Zhang, Lun Cai; *The People’s Hospital of Hejian (81):* Yuan Ma, Zhifeng Ma, Bin Li, Linlin He, Jinghui Li; *Dong'e People’s Hospital (81):* Weibing Zhang, Shuxin Zhang, Hongzhen Zhang, Yingguang Dai; *The First People's Hospital of Shuangliu District, Chengdu (80):* Jun Lei, Lei Mao, Yiyang Huang, Zhi Zhou, Ping Chen; *The First People's Hospital of Longquanyi District, Chengdu (80):* Fang Chen, Pan Wei, Tiangui Li, Honglin Chen, Mengfei Zeng; *Chongqing Bishan District People's Hospital (80):* Kejie Mou, Jun Xue, Yong Jiang; *Affiliated Hospital of North Sichuan Medical College(80):* Xiaoping Tang, Tao Chen, Yalan Zhang, Yanbing Xu, Yuchen Gu; *Tianjin Fifth Central Hospital (80):* Lei Chen, Yujun Zhao, Bin Yang, Peng Kuai, Xi Wang; *Dancheng County People’s Hospital(80):* Yuwang Yang, Xueling Hu, Huitian Zhang, Yintao Yang, Weifeng Wang; *People's Hospital of Ordos Dongsheng District (78):* Junyi Zhang, Wei Cheng, Xiaoxue Zhang, Xiaowen Ma, Qin He; *Zhangjiagang First People's Hospital (74):* Li Zhang, Rong Gao, Huixiang Liu, Jingwei Ye, Ping Xu; *Yantai Yuhuangding Hospital (73):* Xin Wu, Yuan Yuan, Peng Zou, Zhen Zhang, Jiyong Cheng; *Dujiangyan People’s Hospital (70):* Zhangming Zhou, Yijun Zeng, Zhang Liang, Deming Du, Shui Yu; *The Second Affiliated Hospital of Suzhou University (67):* Yongjun Cao, Shoujiang You, Jiaping Xu, Zhichao Huang, Dongqin Chen; Sichuan *Mianyang 404 Hospital (56):* Wenfeng Xiao, Li Zhu, Miao Yuan; *The 904th Hospital of the Joint Logistics Support Force of the Chinese People's Liberation Army (55):* Yuhai Wang, Dongliang Shi, Xu Hu, Dingchao Xiang, Like Shi; *The First Hospital of Shanxi Medical University (53):* Hongqin Wang, Liu Yang, Wang Miao, Yiyi Hu, Yuchun Zhao; *Chongqing Emergency Medical Center (50):* Xi Hu, Yang Liu, Weiduo Zhou, Chao Sun; *Jingzhou Central Hospital (45):* Tao Chen, Dong Tang, Kun Yao, Jin You, Shishi Chen; *Nanhe County People’s Hospital (44):* Jianmin Yao, Huanmei Li, Jinmei Liu, Ailin Bai; *The Second People’s Hospital of Yibin (41):* Yong Yi, Qingshan Deng, Peng Luo, Han Wang, Jingcheng Jiang; *Zhongshan Hospital Affiliated to Xiamen University (40):* Qingwei Yang, Shunpo He; *Pangang Group General Hospital (39):* Jun Wang, Yu Chen, Hua He, Yuyang Deng; *Guangzhou First People’s Hospital (38):* Zhikai Cao, Xuxia Yi, Jinbiao Luo; *Chengdu Fifth People’s Hospital (37):* Shuang Luo, Min Gong, Li Liu, Xuejun Gao, Jia Liu; *The First Affiliated Hospital of Baotou Medical College (36):* Li’e

Wu, Jia Zhang, Hongying Sun, Xinhui Li, Lu Jia; *Yaan People’s Hospital (33):* Jianbing Wu,

Jie Zhang, Huajun Zhang, Chunfu Du, Shun Li; *The First People’s Hospital of Yibin (32):*

Xiaobin Yang, Jie He, Lei Liao; *Jinhua Municipal Central Hospital (31):* Gezhi Zhou, Wentao Dong, Yunxiang Chen, Xiaofeng Lin, Xujian Shui; *No.988 Hospital of Joint Logistic Support Force (31):* Peng Zhang, Yuan Zhao, Hongli Yang, Wenbin Zhao, Xiaoyi Zhang; *Zhongnan Hospital of Wuhan University (31):* Jincao Chen, Qian Wu, Xuan Dai; *Xinghua Traditional Chinese Medical Hospital (29):* Baogui Tang, Yinjuan Wang; *Peking University Third Hospital Yanqing Hospital (28):* Tao Liu, Haixia Zhang; *Wuhan No.1 Hospital (26):* Faliang Duan, Ming Luo; *Shijiazhuang People’s Hospital (25):*Qingfang Jiao, Guoliang Lei, Dong Wang, Chunwang Song, Haopeng Tan; *People's Hospital of Deyang City(24):* Feng Ye, Xinghu Qin, Xiaolong Liang, Junling Liu, Lang Yang; *The First Affiliated Hospital of Chengdu Medical College (22):* Jie Yang, Yapeng Lin, Qian Yang, Xuntai Ma, Yinkuang Qi; *Hebei General Hospital (20):* Baogen Pan, Caixia Jiang, Zhanying Ye, Ce Dong, Xiongfei Yue; *People's Hospital of Xinjiang Uygur Autonomous Region (20):* Xiaopeng Yang, Tuoheti

Maimaitiyiming, Jun Dong, Yonggang Wu, Feng Gao; *Tieling Central Hospital (19):* Deqiang Zhao, Xinghai Zhang; *The First People's Hospital of Yiliang (15):* Pengjun Wang, Hongbo Jiang, Jianping Li, Wei Zhang, Jing Chen; *Shanxi Dayi Hospital (15):* Haibo Tong, Yonghong Wang, Kaipeng Qiao; *The First Affiliated Hospital of Zhengzhou University (12):* Fuyou Guo, Mingchu Zhang, Yan Hu, Mengzhao Feng, Dengpan Song; *No.215 Hospital of Shanxi Nuclear Industry (12):* Yi Zuo, Shangjun Chen, Chao Qian, Baoming Li, Jingku Ma; *The Third People’s Hospital of Chengdu (11):* Sunfu Zhang, Bin Kong, Xingyu Dong, Qiang Li, Sheng Fang; *People's Hospital of Qiandongnan Miao and Dong Autonomous (7):* Bin Lu, Yang Li, Zhen Zhang, Yongling Yang, Hong Yu; *Tiemei General Hospital of Liaoning Province Health Industry Group (7):* Huaiyu Sun, Yue Wang; *Qingdao Municipal Hospital (7):* Weimin Wang, Tong Li, Shengli Li, Zhiming Xu, Yongyi Wang; *Huashan Hospital of Fudan University Neurology Department (7):* Qiang Dong, Yuping Tang, Heling Chu, Ying Lu; *The First Affiliated Hospital of Suzhou University (5):* Zhong Wang, Xiaoou Sun; *The First People's Hospital of Yunnan Province (1):* Jianhua Zhao, Shuaifeng Yang, Xiying Qian.

***India***

*KLES Dr. Prabhakar Kore Hospital & MRC (28):* Aralikatte Saroja, Ravishankar Naik, Sandip Chindhi, Nakul Pampaniya, Kurubara Amaresh; *Government Medical College,*

*Thiruvananthapuram (16):* Thomas Iype, Dileep R, Reeja Rajan, Praveen Panicker; *GNRC*

*Hospital Dispur (12):* Rupjyoti Das, Nupur Choudhury, Pankaja Gohain; *Baptist Christian*

*Hospital, Assam (5):* Jemin Webster, Biyol Pakma, Lalbiak Sangi; *St. Stephen's Hospital, Delhi (4):* Ivy Sebastian, Gaurav Aggrawal, Komal Raj, Deepankshi Rajoura; *Guru Gobind Singh Hospital, Faridkot (2):* Sulena Singh, Varun Aggrawal, Amit Narang.

***Mexico***

*Institituto Nacional de Neurologia y Neurocirugia (9):* Antonio Arauz, Vanessa Cano, Diego

López, Hector Valdéz, Roberto Toledo.

***Nigeria***

*Ahmadu Bello University Teaching Hospital (29):* Reginald Obiako, Sani Abubakar, Oguike

Emeka, Balogun Olayemi, Melika Lois, Ibinaiye Philip, Olurishe Comfort O; *Lagos University Teaching Hospital (26):* Njideka Okubadejo, Osigwe Agabi, Oluwadamilola Ojo; *University of Ilorin Teaching Hospital (15):* Kolawole Wahab, Abiodun Bello, Oyinloye Ibukun, Olufemi Sanayaolu, Sunday Adeniyi, Abdulraheem Jimoh.

***Pakistan***

*Aga Khan University (36):* Mohammad Wasay, Dilshad Begum, Anila Anjum, Shanid Waheed, Ayeesha Kamal; *Shifa International Hospital (12):* Raja Shoiab, Fizza Orooj, Sadaf Majid, Taskeen Zehra Abdus Khan; *South City Hospital (1):* Ravi Shanker, Nadir Syed, Nashwa Ahmad.

***Peru***

*Instituto Nacional de Ciencias Neurológicas (12):* Carlos Abanto, Ana Valencia, Danny

Barrientos, Jorge Ramirez, Pilar Calle.

***Sri Lanka***

*Kurunegala Teaching Hospital (103):* Dilum Palliyeguruge, Sumudu Muthucumarana,

Shiroma Ratnayaka, Dilhara Ganihiarachchi, Arundathi Bandaranayake, S.D.B. Somaratne,

Saumya Narayana, Sithara Gallage; *National Hospital of Sri Lanka (50):* Bimsara Senanayake, Udari Samarasiri, Dunya Luke, Mythily Sivpathasundaram, Vithoosan Sahadevan, Amani Rasmi, Yuran Deshaka; *Gampaha District General Hospital (24):* Nilukshi Fernando, Aruna Munasinghe, Kapilanga Rathnapriya, A.S. Nissanka, Kanchana Karunathilake, Isuru Gayan, Kaminda Wijenayake, Hasitha Gunasekara, Jagath Vidyarathne; *Jaffna Teaching Hospital (22):* Ajantha Keshavaraj, Kanagasabapathy Janarthanan, Arhivalaky Gerald Jeevathasan, Sivaram Sivamainthan, Mathyamuthan John Priyanth, Abirami John Priyanth; *Colombo South Teaching Hospital (16):* Thambippillai Rajendiran, Sanjeewa Alwis, Nushara Gunasekare, Vasundara Liyanarachchi; *Karapitiya Teaching Hospital (11):* Athula Dissanayake, Wimalasiri Uluwattage, Gimhani Ratnayake, Charika Rajinee, Sakura Jayawardana; *Kandy National Hospital (8):* Janaka Peiris, Ranjith Wicramasinghe, Chamila Fernando, Jessie Abbas, Nethmini Withanage, Makaranda Bandara.

***Vietnam***

*Bach Mai Hospital (95):* Duy Ton Mai, Van Chi Nguyen, Viet Phuong Dao, Xuan Trung Vuong, Tien Dung Nguyen, Trung Hieu Dinh, Ha Quan Phan, Quoc Viet Bui, Dinh Tho Phung, Quang ThoPham; *103 Military Hospital (20):* Dinh Dai Pham, Duc Thuan Do, Phuc Duc Dang, Minh Duc Dang, Dang Hai Nguyen; *Thong Nhat Hospital (16):* Thi Phuong Nga Nguyen, Quoc Huy Nguyen, Quoc Dai Pham, Quoc Vinh Chau, Vinh Thy Van Tai; *Thu Duc District Hospital (11):* Tran Vinh Le, Cong Tri Le, Ha Mai Khuong Tran, Huu Khan Nguyen, Hoang Minh Thao Nguyen; *Nguyen Tri Phuong Hospital (10):* Duc Chien Vo, Thai My Phuong Nguyen, Trung Thanh Tran, Thi Hanh Vi Vo, Hao Nhien Cao; *University Medical Hospital (3):* Ba Thang Nguyen, Thi Ngoc Suong Le, Thien Duc La, Chi Duc Pham, Huy Thai.
